# A comprehensive benchmarking study on computational tools for cross-omics label transfer from single-cell RNA to ATAC data

**DOI:** 10.1093/g3journal/jkag026

**Published:** 2026-02-08

**Authors:** Yuge Wang, Hongyu Zhao

**Affiliations:** Department of Biostatistics, Yale School of Public Health, Yale University, New Haven, CT 06511, United States; Department of Biostatistics, Yale School of Public Health, Yale University, New Haven, CT 06511, United States; Program of Computational Biology and Bioinformatics, Yale University, New Haven, CT 06511, United States; Department of Genetics, Yale School of Medicine, Yale University, New Haven, CT 06511, United States

**Keywords:** scATAC-seq, scRNA-seq, cross-omics label transfer, cell type annotation, benchmarking study

## Abstract

With continuous progress of single-cell chromatin accessibility profiling techniques, scATAC-seq has become more commonly used in investigating regulatory genomic regions and their involvement in developmental, evolutionary, and disease-related processes. At the same time, accurate cell type annotation plays a crucial role in comprehending the cellular makeup of complex tissues and uncovering novel cell types. Unfortunately, the majority of existing methods primarily focus on label transfer within scRNA-seq datasets and only a limited number of approaches have been specifically developed for transferring labels from scRNA-seq to scATAC-seq data. Moreover, many methods have been published for the joint embedding of data from the two modalities, which can be used for label transfer by adding a classifier trained on the latent space. Given these available methods, this study presents a comprehensive benchmarking study evaluating 27 computational tools for scATAC-seq label annotations through tasks involving single-cell RNA and ATAC data from various human and mouse tissues. We found that when high-quality paired data were available to transfer labels across unpaired data, Bridge and GLUE were the best performers; otherwise, bindSC and GLUE achieved the highest prediction accuracy overall. All these methods were able to use peak-level information instead of purely relying on the gene activities from scATAC-seq. Furthermore, we found that data imbalance, cross-omics dissimilarity on common cell types, data binarization, and the introduction of semi-supervised strategy usually had negative impacts on model performance. In terms of scalability, we found that the most time and memory efficient methods were Bridge and deep learning-based algorithms like GLUE. Based on the results of this study, we provide several suggestions for future methodology development.

## Teaser

This article targets researchers interested in cell type annotation using single-cell data. The study presents a systematic comparison of computational tools for transferring labels from single-cell RNA sequencing data to single-cell ATAC sequencing data. The authors evaluated 27 methods using human and mouse datasets and assessed annotation accuracy, robustness under different data conditions, and computational efficiency. The results identify methods that perform best with or without paired RNA and ATAC data and highlight factors that reduce performance. These findings support informed method selection and guide future development of cross-omics single-cell annotation methods.

## Introduction

The integration of single-cell RNA sequencing (scRNA-seq) and single-cell assay for transposase-accessible chromatin sequencing (scATAC-seq) has become instrumental in unraveling the intricacies of cellular heterogeneity and regulatory mechanisms at a single-cell resolution (Buenrostro et al. 2018; Fiers et al. 2018; Jia et al. 2018; Packer and Trapnell 2018; Carter and Zhao 2021). However, the accurate annotation of scATAC-seq cells remains a challenge and involves a combination of automated annotations from computational tools and subsequent manual corrections (Clarke et al. 2021). While numerous tools exist for annotating cell types in scRNA-seq data (Abdelaal et al. 2019; Pasquini et al. 2021), the options tailored specifically for scATAC-seq data are limited. In addition, many methods have been designed for computational integration of scRNA-seq and scATAC-seq data, typically resulting in a joint embedding of cells from both modalities in a low-dimensional space (Argelaguet et al. 2021; Miao et al. 2021a; Xu and McCord 2022). Once this joint embedding is obtained, label transfer can be easily conducted such as through an external k nearest neighbor (kNN) classifier trained on the latent space. As scATAC-seq matures and becomes widely adopted in single-cell studies, it is crucial to conduct a comprehensive evaluation of the performance of methods from both categories in annotating scATAC-seq data.

Within the first category, there are two different types of annotation tools applicable for the annotation of scATAC-seq data. The first type comprises tools initially designed for scRNA-seq data (intra-modality annotation), while the second type includes tools specifically designed for scATAC-seq data (cross-modality annotation). Popular methods in the first type are Seurat (Stuart et al. 2019), Conos (Barkas et al. 2019), and scGCN (Song et al. 2021) and two representative methods in the second type include scJoint (Lin et al. 2022) and Bridge (Hao et al. 2024). In contrast to other methods that directly transfer labels from scRNA-seq to scATAC-seq after consolidating the feature set through gene activity calculation, Bridge utilizes multimodal data as a bridge. This approach helps circumvent potential information loss and inaccuracies in assumptions about feature relationships between the two modalities.

For the second category, there are also two types of tools that can perform joint embedding of scRNA-seq and scATAC-seq data. For tools belonging to the first type, they were originally designed for integrating different scRNA-seq data with batch effects, such as LIGER (Welch et al. 2019), scVI (Lopez et al. 2018), SCALEX (Xiong et al. 2022), and scDML (Yu et al. 2023). These methods can be applied to integrate scRNA-seq and scATAC-seq data once the ATAC data are transformed to gene activities. The second type includes tools that were published more recently and designed for integrating multimodal single-cell data. Although methods under this type advertised themselves for the ability to integrate scRNA-seq and scATAC-seq data, some of them still require gene activity calculation for the ATAC data in order to directly align the feature sets of two modalities, such as cross-modal AE (Yang et al. 2021), uniPort (Cao et al. 2022), and scMC (Zhang and Nie 2021). There are also other methods that can utilize the ATAC peak data such as MMD-MA (Liu et al. 2019), UnionCom (Cao et al. 2020b), SCOT (Demetci et al. 2022a, 2022b), Pamona (Cao et al. 2021), MultiMAP (Jain et al. 2021), scDART (Zhang et al. 2022), GLUE (Cao and Gao 2022), bindSC (Dou et al. 2022), UINMF (Kriebel and Welch 2022), MultiVI (Ashuach et al. 2023), Cobolt (Gong et al. 2021), and StabMap (Ghazanfar et al. 2024), among which MultiVI and Cobolt work like Bridge that require an additional paired data to integrate unpaired RNA and ATAC data. GLUE and StabMap have two versions of themselves. One version does not require paired data and the other version can use paired data to help better integrate unpaired data if available.

Previously, we published a benchmarking study on this topic on a smaller scale, using only five datasets and focusing solely on the five methods from the first category (Wang et al. 2022). This current study represents a substantial expansion, encompassing all the 27 computational tools mentioned above. We employed a more extensive selection of single-cell RNA and ATAC data from both human and mouse tissues, each with available cell type annotations serving as the ground truth. The data we collected included nine paired data (multimodal) where scATAC-seq and scRNA-seq were simultaneously measured in each single cell and 18 unpaired data (unimodal) where scATAC-seq and scRNA-seq were separately measured from the same tissue. Among the unpaired samples, nine had corresponding paired data from the same species and tissue, facilitating the evaluation of methods that require additional paired data for direct label transfer from RNA to ATAC or integration of data from the two modalities. We evaluated the performance of different methods on both annotation accuracy and scalability. For accuracy, we considered both accuracy metrics calculated on common cell types and metrics calculated on ATAC-specific cell types. For scalability, we compared running time and peak memory usage across a wide range of data sizes.

Beyond benchmarking and ranking, we performed additional comparisons and designed experiments to understand how model performance is affected by different data properties and method properties. For data properties, we considered three aspects, namely the proportion of modality-specific cell types, the discrepancy in the common cell type compositions between modalities, and the cross-omics dissimilarity of common cell types. For method properties, we discussed how the incorporation of paired data, peak level information of ATAC data, and the introduction of semi-supervised training would affect label transfer accuracy. At the end of this paper, we extensively discuss observations related to both method and data properties and provide guidance for users in selecting or developing suitable tools for cell type annotation in scATAC-seq data.

## Materials and methods

### Single-cell data preprocessing

A full list of data used in this study can be found in the Supplementary Table 1 in File “Supp Tables.xlsx.” For each tissue, it can contain at most three types of datasets, namely unimodal RNA data, unimodal ATAC data, and multimodal data (RNA and ATAC measured simultaneously for the same cell). To facilitate the evaluation of label prediction performance, we manually unified the naming conventions of cell labels provided in the scRNA-seq and scATAC-seq. We also removed cell types from each dataset that contained less than 10 cells. As a common practice, for all the scATAC-seq data (both unimodal and multimodal), if the raw gene activity matrix in integers was not available, we calculated it using function “GeneActivity” in R package Signac with the fragment files as inputs (Stuart et al. 2021). For all the tissues that had all types of data, we were able to use them to benchmark methods that could integrate paired and unpaired data together, including Bridge, MultiVI, Cobolt, GLUE (multi), and StabMap (multi). Therefore, we had to align the peak set of unimodal ATAC and that of the multimodal ATAC data. If the fragment files of the multimodal ATAC data were available, we requantified the abundance of multimodal ATAC peaks using the unimodal peak set as reference by the “FeatureMatrix” function in Signac. As the last step of data processing, if a dataset contained more than 20k cells, we downsampled it proportionally to contain 20k cells except for the cell types that originally contained less than 100 cells. Descriptions of preprocessing pipelines specific to each dataset, like exceptions to the common practices mentioned above, are provided below. Details for data preprocessing can be found in our GitHub repository. Tissues that only have unimodal data (unpaired) or multimodal data (paired) are indicated correspondingly in the following paragraphs. Processing details for each dataset can be found in Supplementary Text 1 in File “Supp Text.doc.”

### Description and implementation of methods

There are two categories of methods we benchmarked in this study, methods that are designed for label transfer and methods that are originally designed for scRNA-seq and scATAC-seq joint embedding. The description of all computational methods we can be found in Supplementary Text 2 in File “Supp Text.doc.”

For all the joint embedding methods that do not explicitly perform label transfer, we first obtained the latent representations of both scRNA-seq and scATAC-seq data using the default or recommend number of latent dimensions for each method. When no default or recommended number is available, we used 20. Then, a kNN classifier (*k* = 30) was trained using the latent representations and known cell labels of scRNA-seq data. Last, we used the trained kNN classifier to get the predicted probability matrix for the corresponding scATAC-seq data for all downstream evaluations.

Note that for StabMap (base version without paired data being used), scDART and GLUE, they all require the region-to-gene relationships to link ATAC peaks and RNA genes as prior information. We used GLUE's Python function “scglue.genomics.rna_anchored_guidance_graph” to obtain the required format of region-to-gene relationships for all the three methods. Since the reference genome is needed when running this function, we used the genome version indicated in the original publications of each scATAC-seq dataset (shown in Supplementary Table 1 in File “Supp Tables.xlsx”).

For methods that do not require additional paired multi-omics data, we used the raw count matrix of scRNA-seq and raw count matrix or gene activity matrix or both of scATAC-seq data based on the requirements of each method (see Table 1) as inputs. For methods that require paired data to integrate or transfer labels from unpaired RNA data to ATAC data, we provided raw count matrices of scRNA-seq, scATAC-seq and multimodal data (with the peak sets of unimodal and multimodal ATAC data aligned). The implementation of each method followed the instructions on their websites or in their original publications. Details can be found in the scripts on our GitHub repository and software versions can be found in Supplementary Table 3 in File “Supp Tables.xlsx.”

**Table 1.** Overview of methods considered in this benchmarking study.

	First online (m/y)	Main idea^a^	Default Latent dim	Gene activity used	ATAC peak used	Paired data needed	Deal with data-specific structure	Semi-supervised	GPU	Soft-ware	Ref
Bridge	02/22	Dictionary learning	—	X	√	√	√	X	X	R	Hao et al. (2024)
Conos	11/18	Cell graph	—	√	X	X	√	X	X	R	Barkas et al. (2019)
Seurat	11/18	CCA	—	√	√	X	√	X	X	R	Stuart et al. (2019)
scGCN	09/20	GCN	—	√	X	X	√	√	√	Python	Song et al. (2021)
scJoint	04/21	NN	64	√	X	X	√	√	√	Python	Lin et al. (2022)
MMD-MA^b^	05/19	Kernel space matching	5	X	√	X	X	X	√	Python	Liu et al. (2019)
UnionCom	02/20	Metric space matching	32	X	√	X	X	X	√	Python	Cao et al. (2020b)
SCOT											
v1	11/20	OT	Input	X	√	X	X	X	X	Python	Demetci et al. (2022a)
v2^b^	11/21		10				√				Demetci et al. (2022b)
Pamona	11/20	OT	30	X	√	X	√	X	X	Python	Cao et al. (2021)
MultiMAP	03/21	UMAP	2	√	√	X	X	X	X	Python	Jain et al. (2021)
scVI	03/18	VAE	None^c^	√	X	X	X	X	√	Python	Lopez et al. (2018)
Cross-modal AE	12/19	AE	128	√	X	X	X	X	√	Python	Yang et al. (2021)
SCALEX	10/21	VAE	10	√	X	X	X	X	√	Python	Xiong et al. (2022)
scDML	09/22	Metric learning	32	√	X	X	X	X	√	Python	Yu et al. (2023)
uniPort	02/22	VAE + OT	16	X/√^d^	√/X^d^	X	√	X	√	Python	Cao et al. (2022)
scDART	01/22	NN	8	X	√	X	X	X	√	Python	Zhang et al. (2022)
GLUE											
base	09/21	Graph VAE	50	X	√	X	√	X	√	Python	Cao and Gao (2022)
multi						√					
scMC	01/21^e^	Variance analysis	40	√	X	X	X	X	X	R	Zhang and Nie (2021)
bindSC	12/20	CCA	None^c^	√	√	X	X	X	X	R	Dou et al. (2022)
LIGER	11/18	NMF	20-40^f^	√	X	X	√	X	X	R	Welch et al. (2019)
UINMF	04/21	NMF	30-40^f^	√	√	X	√	X	X	R	Kriebel and Welch (2022)
MultiVI^b^	09/21	VAE	None^c^	X	√	√	X	X	√	Python	Ashuach et al. (2023)
Cobolt	11/21	VAE	None^g^	X	√	√	X	X	√	Python	Gong et al. (2021)
StabMap											
base	02/22	Mosaic data integration	50	X	√	X	X	opti-onal	X	R	Ghazanfar et al. (2024)
multi						√					

^a^Full names of abbreviations shown in this column: CCA: canonical correlation analysis; GCN: graph convolutional network; NN: neural networks; OT: optimal transport; UMAP: uniform manifold approximation and projection; AE: autoencoder; VAE: variational autoencoder; NMF: non-negative matrix factorization.

^b^MMD-MA, SCOT (v2) and MultiVI are the only three methods that have not been published in any peer-reviewed journals.

^c^For bindSC, scVI and MultiVI, there is not default or recommended number of latent dimensions. We used 20 for them in this benchmarking study.

^d^For uniPort, there are two choices for the inputs of scATAC-seq data, one is the original peak data and the other is the gene activity matrix. The gene activity calculation is not required because it utilizes OT but is recommended to be used for better data integration performance (Cao et al. 2022). Therefore, in our study, gene activity matrices were used to run uniPort.

^e^For scMC, we could not find a preprint before it was published on Genome Biology, so the date here is the online time publication date.

^f^For LIGER and UINMF, there is no default number of latent space. The ranges shown in the table are the recommend range for choice of latent dimensions. In this study, 20 and 30 were used for LIGER and UINMF respectively.

^g^For Cobolt, there is not default number of latent dimensions. We used 10 for this benchmarking study as this is the number recommended in their online tutorials.

### Evaluation metrics

We assessed the performance of different cross-omics label transfer methods from two aspects, namely accuracy and scalability. For prediction accuracy evaluation, we divided ATAC cells into two parts and designed metrics for them separately: (1) cells whose cell types also existed in the corresponding scRNA-seq data and we call these common cells; (2) cells whose cell types were only observed in scATAC-seq data and we call these ATAC-specific cells. For scalability evaluation, we compared both the running time and memory usage among all methods across selected gradients of sample sizes (see details in 2.4 Benchmarking design). All methods were run on Yale's high performance computing clusters with one computing core.

For common cells, we evaluated performance using balanced accuracy, overall accuracy, weighted accuracy, and macro F1. For ATAC-specific cells, we used weighted accuracy, entropy, enrichment, and the F1 scores of entropy and enrichment. Detailed descriptions of how these metrics including were calculated are provided in Supplementary Text 3 of the File “Supp Text.doc.”

### Benchmarking design

In the following paragraphs, we will describe in detail the benchmarking design for experiments conducted in this study.

*General performance comparison across different tissues*. We ran all 27 methods, including 22 methods for unpaired data and 5 methods that involved additional paired data, on 27 dataset combinations, including 18 unpaired data (9 with available paired data from the same tissue) and 9 paired data. For the nine paired datasets, they were used as if the RNA and the ATAC data were sequenced separately. Therefore, we ran in total of 639 method and data combinations (22*27 + 5*9) to assess the general performance of all selected methods across different tissues.

*Impact of cell type imbalance between modalities*. The original RNA and ATAC data could contain different cell types, and the compositions of cell types could vary significantly as well. We studied the impact of cell type imbalance between the two modalities on model performance; we manually subsampled the RNA and ATAC data for each tissue in two ways. First, we deleted all modality-specific cell types for each tissue to make the RNA and ATAC data contain the same set of cell types. We called this common cell type experiment. Second, apart from deleting modality specific cell types, we downsampled cells to make sure that the numbers of cells that belong to the same cell types are the same between RNA and ATAC. We called this balanced cell type experiment.

*Impact of data binarization*. We investigated whether data binarization could affect the performance of any methods across datasets we collected by binarizing both the RNA data and the ATAC data first before inputting them into the algorithms.

*Effect of semi-supervised strategy*. To investigate the effect of introducing semi-supervised training strategy to deep-learning-based methods on the performance of cross-omics label transfer. We downloaded and modified the original code of selected methods, including scDART, scDML, SALEX, cross-modal AE, GLUE, uniPort, scVI, MultiVI, and Cobolt. Specifically, we connected the latent layer of each method with an additional linear layer whose number of nodes was equal to the number of clusters in the RNA data and added a cross-entropy loss calculated using the RNA data only to the original loss function. For scJoint, since it already employed the semi-supervised training strategy, we removed the cross-entropy term from its loss function to obtain an unsupervised version of scJoint.

*Time and memory usage comparison*. To study the scalability of all 27 methods, we used the mouse MOp data to measure the running time and peak memory usage across seven data sizes, including 1k, 3k, 5k, 10k, 15k, 20k, and 50k cells.

## Results

### Cross-omics label transfer benchmarking design

**Fig. 1.**
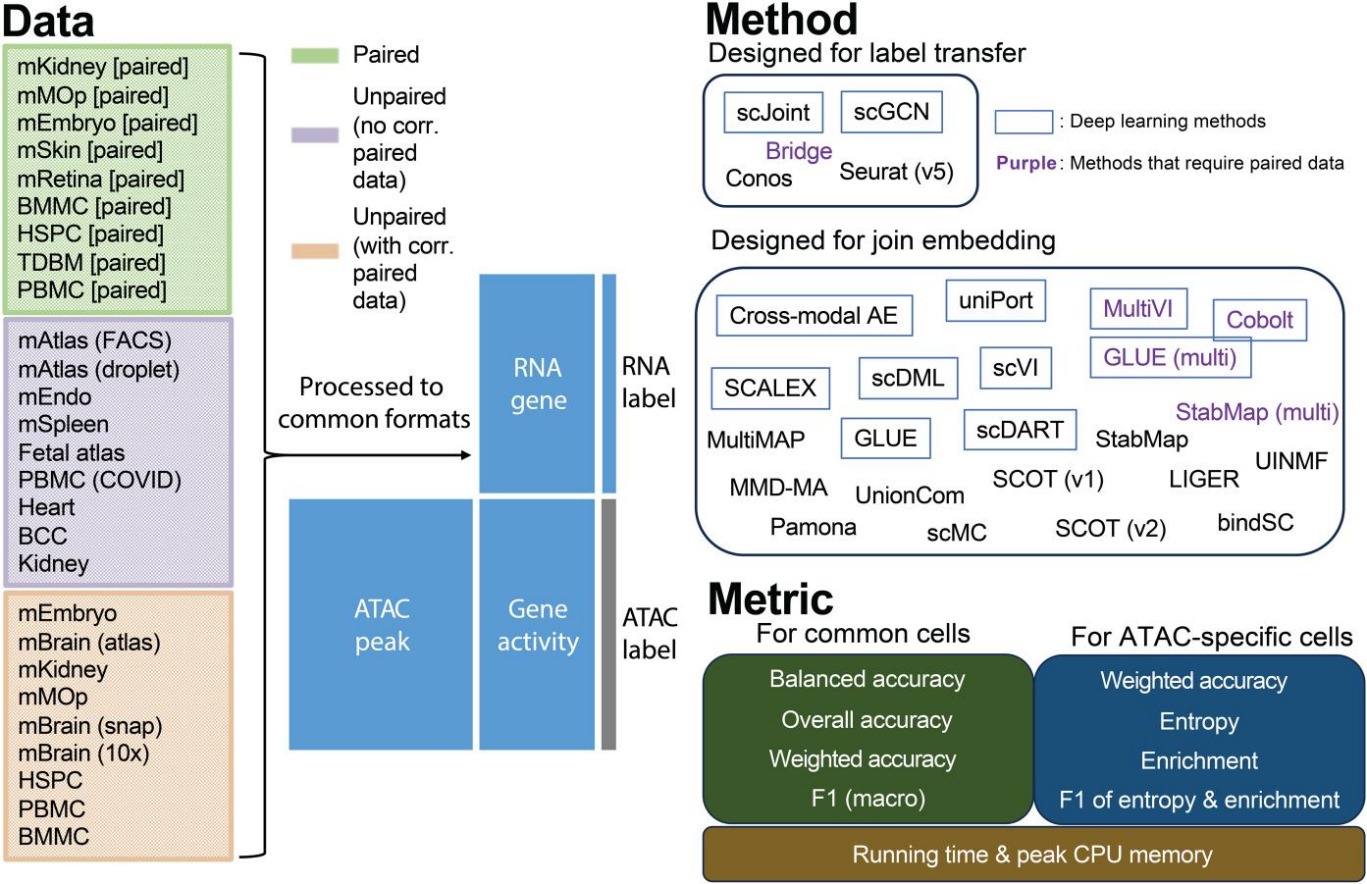
Overview of the benchmarking design in terms of data, methods, and metrics. In this study, we evaluated 27 methods, where five methods are designed for label transfer which means that the predicted labels are included in the outputs of these algorithms, and the other 22 methods are designed for joint embedding of scRNA-seq and scATAC-seq data. To perform label transfer, we first obtained the joint embedding and then used that to train a kNN classifier (*k* = 30). Among both categories of methods, there are in total five methods that involve additional paired data, so they were only run on the third group of datasets, where unpaired RNA and ATAC data had corresponding paired data. The full details about the 27 methods can be found in Table 1 and Supplementary Text 2 in File “Supp Text.doc.”

Here, we provide a comprehensive and quantitative evaluation of 27 computational tools for cross-omics label transfer from scRNA-seq data to scATAC-seq data. The overview of our benchmarking design is shown in Fig. 1.

We collected a variety of scRNA-seq and scATAC-seq datasets from both human and mouse tissues, including brain (Saunders et al. 2018; Chen et al. 2019; Fang et al. 2021), primary motor cortex (Yao et al. 2021), retina (Dou et al. 2022), skin (Ma et al. 2020), kidney (Cao et al. 2018; Muto et al. 2021; Miao et al. 2021b), spleen (Chen et al. 2018; Jain et al. 2021), blood (Granja et al. 2019; Wilk et al. 2021), bone marrow (Buenrostro et al. 2018; Pellin et al. 2019; Luecken et al. 2021; Persad et al. 2023), arteries (Zhu et al. 2020), heart (Hocker et al. 2021), embryo (Pijuan-Sala et al. 2019; Pijuan-Sala et al. 2020; Argelaguet et al. 2022), and basal cell carcinoma (Satpathy et al. 2019; Yost et al. 2019). Apart from tissue-specific data, we also collected a mouse atlas (Cusanovich et al. 2018; Tabula Muris Consortium et al. 2018) and a human fetal atlas dataset (Domcke et al. 2020; Cao et al. 2020a). All data can be divided into three groups: paired data, unpaired data with no corresponding paired data from the same tissue, and unpaired data with corresponding paired data from the same tissue. For the paired data, we did not use the pairing information when applying each method. For the unpaired data with corresponding paired data, the pairing information of the paired data was used for methods that can integrate both unpaired and paired data. Before running all methods, we processed all data to common formats, which are RNA gene expression count matrix, ATAC peak count matrix, ATAC gene activity matrix, RNA cell type labels, and ATAC cell type labels. The ATAC cell type labels were only used for evaluation. The full details about the data we collected and the steps we used to process the data can be found in Supplementary Table 1 in File “Supp Tables.xlsx” and Materials and Methods (2.1).

In addition to the 27 methods, we used kNN and random classifiers as the baseline competitors. For kNN classifiers, all common features between scRNA-seq and gene activity matrix calculated from the scATAC-seq data were used for training. For random classifiers, labels were predicted based on the background probabilities of cell types in the scRNA-seq data.

After getting the predicted probability matrix from each method for ATAC cells, we assessed the prediction accuracy for common cells and ATAC-specific cells separately. For common cells, we calculated four metrics, namely balanced accuracy, overall accuracy, weighted accuracy, and F1 (macro) of precision and recall. For the first, second, and fourth metrics, they were calculated based on the predicted label of each cell, which was the cell type whose predicted probability was the highest. For weighted accuracy, we considered the similarity among cell types by calculating the weighted average of the entire predicted probability vector of each cell. Therefore, even though a predicted label was false, the score could be high if similar cell types had higher predicted probabilities. For ATAC-specific cells, we calculated four metrics as well, namely weighted accuracy, entropy score, enrichment score, and F1 of entropy and enrichment. In addition to accuracy evaluation, we measured the scalability of each method through the running time and peak CPU memory usage across selected gradients of sample sizes. Details about the metrics we used can be found in Materials and Methods (2.3).

In addition to assessing the overall performance of the 27 methods across the 27 datasets (tasks), we conducted an in-depth analysis to explore factors that could have played important roles in cross-modality label transfer from the aspects of both data properties and model properties.

### Performance across different tissues

**Fig. 2.**
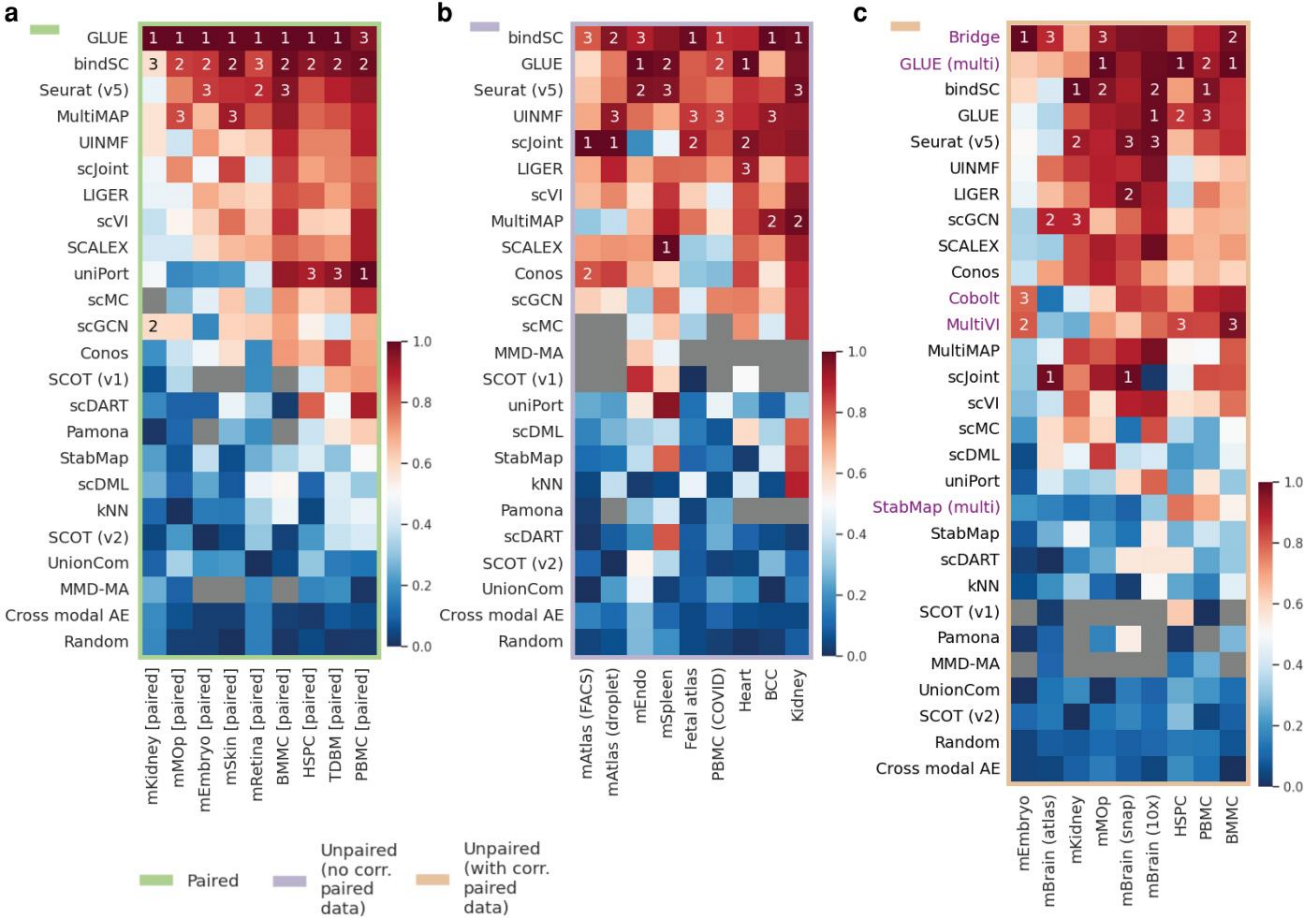
Performance of 27 methods on cross-omics label transfer (RNA to ATAC) across 27 mouse and human datasets. Datasets were divided into three groups: paired datasets (pairing information not used), unpaired datasets without corresponding paired data available, and unpaired datasets with corresponding paired data available. “Corresponding” means from the same tissue and species. (a) The average of four min-max scaled metrics evaluated on common cells (balanced accuracy, overall accuracy, weighted accuracy and F1 [macro]) for paired datasets. (b) The average of four min-max scaled metrics evaluated on common cells for unpaired datasets with no corresponding paired data. (c) The average of four min-max scaled metrics evaluated on common cells for unpaired datasets with corresponding paired data. For each heatmap, methods (rows) are placed from top to bottom in the descending order of their averaged values across all datasets (columns) in that heatmap and methods ranked top three in each dataset are marked by Arabic numbers. Heatmaps for each metric in their original scales, including those for common cells and for ATAC-specific cell, can be found in Supplementary Fig. 2–4 in File “Supp Figures.doc.”

To equally weigh the contribution of different metrics and tasks to the performance comparison among different methods, we min-max scaled the values of each metric within a task to make sure that the scaled metric covered the entire range from 0 to 1 for a task. The overall prediction accuracy on common cell types for each task and method combination was calculated by averaging across all the four min-max scaled metrics. Results for each of the three groups of tasks (datasets) mentioned previously are shown in Fig. 2. For the original unscaled metrics, they can be found in Supplementary Fig. 2–4 in File “Supp Figures.doc.”

For paired data (Fig. 2a), GLUE, bindSC, and Seurat (v5) performed the best, followed by MultiMAP and UINMF. GLUE and bindSC were the only two methods that consistently showed up in the top three performers across all tasks. Especially for GLUE, it was the best performer in eight out of nine tasks. The performance of uniPort seemed to vary depend on the specific task. Among the nine tasks, it was among the top three performers in three of them.

For unpaired data without corresponding paired data available (Fig. 2b), the top three performers were bindSC, GLUE, and Seurat (v5), with UINMF and scJoint following closely behind when considering the metrics calculated on common cells. In terms of F1 of entropy and enrichment calculated on ATAC-specific cells (Supplementary Fig. 3b in File “Supp Figures.doc”), random classifiers undoubtedly achieved the highest scores, followed by scGCN and uniPort, indicating that these two methods performed most similar to a random classifier. Moreover, we observed that scJoint had very low F1 of entropy and enrichment and relatively high weighted accuracy on ATAC-specific cells. This suggests that scJoint tended to classify ATAC-specific cell types to existing cell types that were biologically similar to them.

For unpaired data with corresponding paired data available (Fig. 2c), we were able to assess all the 27 methods including the five methods that required paired data as a “bridge” to transfer information across omics. For metrics calculated using common cells, the top three performers were Bridge, GLUE (multi), and bindSC, followed by GLUE and Seurat (v5). Regarding metrics computed with ATAC-specific cells (Supplementary Fig. 4b in File “Supp Figures.doc”), methods that performed most akin to a random classifier were scGCN and scDML. As for scJoint, similar to what was observed in the second type of datasets, it achieved relatively low F1 of entropy and enrichment and high weighted accuracy on ATAC-specific cells.

Note that for LIGER, there were two options when selecting highly variable genes (HVGs), which was one of the steps of the algorithm used in LIGER. One option was selecting using both the RNA and ATAC data and the other was only using the RNA data. We found there were no significant differences between these two versions of LIGER across all metrics (Supplementary Fig. 5a in File “Supp Figures.doc”), so we used the first version of LIGER throughout the entire paper. Like LIGER, StabMap also had two options when selecting the reference space to map onto. We found that when using ATAC as the reference, seven out of eight metrics achieved higher values than using RNA as the reference (Supplementary Fig. 5b in File “Supp Figures.doc”). Therefore, we used ATAC as the reference space for StabMap in our study.

### The impact of data properties

**Fig. 3.**
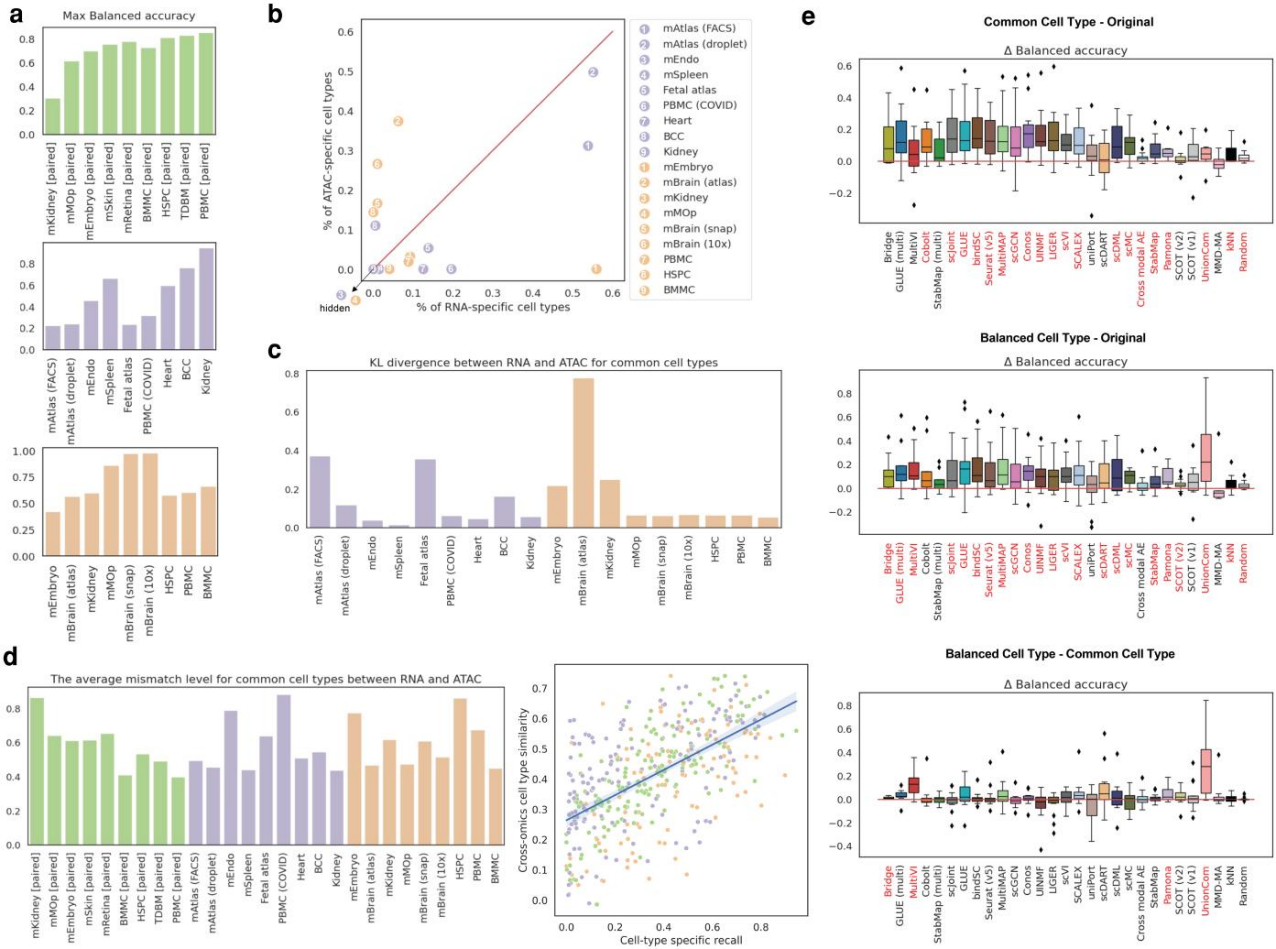
The impact of data properties, including data imbalance and cross-omics cell type similarity, on prediction accuracy. (a) The maximum values of balanced accuracy achieved on each dataset across all the methods. Information of other metrics can be found in Supplementary Fig. 1 in File “Supp Figures.doc.” (b) The proportions of modality-specific cell types for unpaired datasets. (c) The discrepancy in cell type compositions between two modalities for unpaired datasets measured by KL divergence. (d) The influence of cross-omics mismatch level on prediction accuracy. On the left is the cross-omics mismatch level, which is defined as 1 minus the cross-omics similarity, averaged across all common cell types between RNA and ATAC for each task. On the right is the relationship between cross-omics cell type similarity and cell-type specific recall. Each dot is the average recall across all methods for a specific common cell type in one dataset. The cross-omics similarity for each common cell type was calculated by the Pearson correlation using 2,000 common HVGs. (e) The impact of manually removing cell type imbalance on balanced accuracy. “Common cell type” means modality-specific cell types were removed and “balanced cell type” means cell type compositions were made the same between two modalities. Pairwise comparisons among these two cases and the original case for each method and dataset combination were performed. For all boxplots, methods marked in red along the *x*-axes are methods that achieved significant positive differences (*P*-value threshold: 0.05; statistical test: one-sample *t*-test). The impact on other metrics can be found in Supplementary Fig. 6–8 in File “Supp Figures.doc.”

Previously, we min-max scaled each metric to equally weigh the contributions of different metrics to the overall prediction accuracy. However, if we take a look at the metrics in their original scales, we will find for some tasks, even the best performers had a very low prediction accuracy (Fig. 3a and Supplementary Fig. 1 in File “Supp Figures.doc”). For example, for mKidney [paired], mAtlas (FACS), mAtlas (droplet), fetal atlas, and PBMC (COVID), the maximum balanced accuracy was only around 0.2–0.3. We found this was due to the properties of the datasets used in the task, including the level of data imbalance and cross-omics cell type dissimilarity (Fig. 3).

#### Data imbalance

There were two levels of data imbalance: the proportion of modality-specific cell types (Fig. 3b) and the discrepancy in the compositions of common cell types (Fig. 3c). We found that datasets with either high proportions of modality-specific cell types or substantial discrepancies in common cell type compositions exhibited poor performance across all methods. For example, mAtlas (FACS), mAtalas (droplet), and mEmbryo contained over 50% of RNA-specific cell types. The discrepancy in common cell type compositions of fetal atlas and mBrain (atlas) ranked the highest among all human datasets and all mouse datasets, respectively.

In addition to the aforementioned empirical evidence, we studied the impact of cell type imbalance on prediction accuracy by manually adjusting data imbalance at the two levels. First, we equalized the sets of cell types between the two modalities by removing modality-specific cell types from both training and evaluation, denoting this as the “common cell type” experiment. Second, we further manually selected cells to ensure that RNA and ATAC data had the same number of cells for each common cell type and referred to as the “balanced cell type” experiment.

When comparing the “common cell type” with the original data (Fig. 3e top and Supplementary Fig. 6 in File “Supp Figures.doc”), we observed that 18 out of the 27 methods (66.7%) demonstrated increased prediction accuracy for at least one metric assessed on common cells. This suggests that merely removing modality-specific cell types could significantly improve the prediction accuracy for common cells. In the comparison of the “balanced cell type” with the original data (Fig. 3e middle and Supplementary Fig. 7 in File “Supp Figures.doc”), we noted an increase in the number of methods that had improved prediction accuracy (22 out of 27; 81.5%). The methods whose performance remained unaffected in both experiments were uniPort, SCOT (v1), and MMD-MA. Moreover, we investigated whether balancing cell type compositions could improve the prediction accuracy beyond the removal of modality-specific cell types by directly comparing the results of the “balanced cell type” with the results of the “common cell type” experiment (Fig. 3e bottom and Supplementary Fig. 8 in File “Supp Figures.doc”). This time, fewer methods showed significant improvement in at least one metric (14 out of 27; 51.9%). Especially for balanced accuracy, significant changes were only observed for Bridge, MultiVI, Pamona, and UnionCom, with changes being non-negligible for only MultiVI and UnionCom.

#### Cross-omics cell type dissimilarity

Although some datasets did not exhibit high data imbalance, their cross-omics cell type dissimilarity was very high, contributing to their poor label transfer performance across all the methods. For example, mEndo and PBMC (COVID) were the tasks that had the highest mismatch scores for common cell types between RNA and ATAC among all unpaired mouse and human tasks, respectively. So as for mKidney [paired] in the paired data group. Overall, we found that the cross-omics similarity for common cell types was positively related with the prediction accuracy (Fig. 3d right, correlation: 0.59, *P*-value: 6.7e-40).

We noted that different methods processed single-cell ATAC data differently. For example, scDART, MultiVI, and StabMap binarized the ATAC peak matrix and scJoint even binarized both the RNA and ATAC gene matrices. According to scJoint, data binarization of both RNA and ATAC improved its performance because the distributions of two modalities were made more similar. To investigate whether this also applied to other methods, we performed data binarization before running each method. From Supplementary Fig. 9 in File “Supp Figures.doc,” we first observed that data binarization had minimal effect on scJoint as expected. Then, we found none of the methods benefitted from data binarization in terms of the prediction accuracy on common cells and 14 methods out of 27 (51.9%) had significantly decreased accuracy in at least one metric. The methods that were affected most by data binarization were UINMF and LIGER. Interestingly, for the metrics calculated on ATAC-specific cells, we found the weighted accuracy was only significantly and negatively affected for Bridge and the F1 of entropy and enrichment was positively affected for 13 methods, among which 10 methods' prediction accuracy on common cells were negatively affected. The methods that were not affected by data binarization at all were GLUE (multi), MultiVI, Cobolt, scJoint, MultiMAP, scDART, scDML, SCOT (v1), and UnionCom.

### The impact of method properties

#### The involvement of paired data

**Fig. 4.**
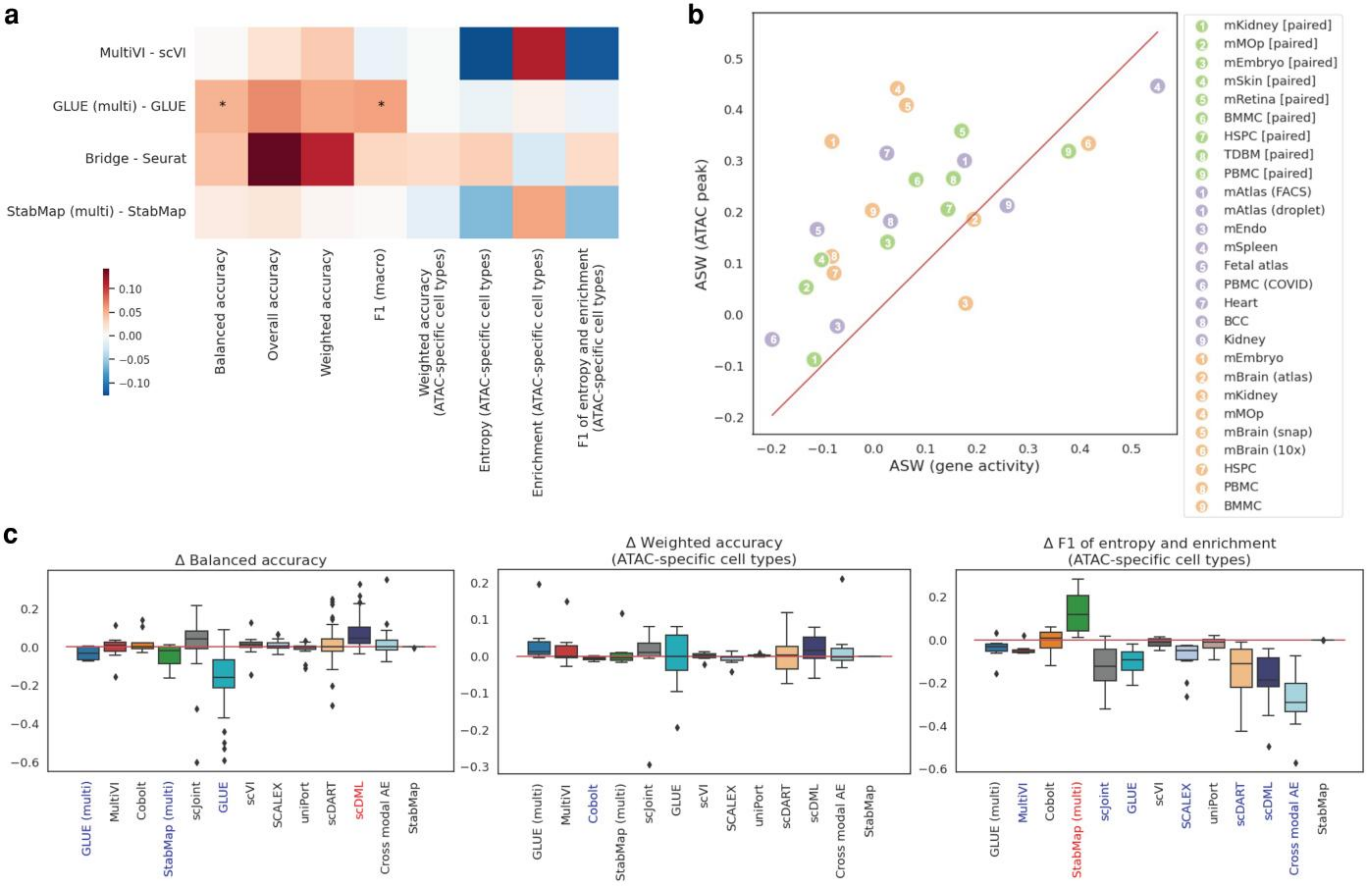
The impact of method properties, including the usage of paired data, the usage of original ATAC peak data, and the introduction of semi-supervised strategy. (a) The impact of using paired data as a “bridge.” The heatmap shows the differences in accuracy metrics (columns) averaged across all tissues with asterisks indicating statistical significance. (b) The comparison of average Silhouette width (ASW) between UMAPs drawn using ATAC peak data and gene activity (GA) matrix. Note that we have one less ATAC data than the total number of datasets because mAtlas (FACS) and mAtlas (droplet) shared the same ATAC data. (c) The impact of introducing semi-supervised strategy to 13 selected methods on prediction accuracy. The impact was evaluated by the differences between metrics calculated using results after and before employing the semi-supervised strategy. From left to right, the three boxplots show the differences in balanced accuracy assessed on all common cells, weighted accuracy and F1 of entropy and enrichment accuracy assessed on ATAC-specific cells. Methods marked in red and blue along the *x*-axes are methods that achieved positive and negative significant differences respectively. All statistical tests conducted here were one-sample *t*-tests with *P*-value threshold being 0.05.

Since we found both of the top two performers for common cells' prediction accuracy in Fig. 2c were methods that involved paired data as a “bridge,” we wanted to study how using additional paired data could affect the label transfer accuracy by directly comparing four pairs of methods: MultiVI and scVI, GLUE (multi) and GLUE, Bridge and Seurat, and StabMap (multi) and StabMap (Fig. 4a and Supplementary Fig. 10 in File “Supp Figures.doc”). We found the prediction accuracy on common cells was significantly improved for GLUE (multi) and directionally positive for Bridge, while the metrics for ATAC-specific cells did not change much. For MultiVI and StabMap (multi), their performance did not change significantly compared to their base versions, respectively.

#### The utilization of the peak information

All the top performers mentioned in “Performance across different tissues” section were methods that utilized the ATAC peak-level information in some way. This is likely because the peak-level data contained more biological information than the gene-level data for scATAC-seq. Specifically, we found different cell types had better separations in UMAPs derived from the peak data compared to that from the gene activities (Supplementary Fig. 11 in File “Supp Figures.doc”) and the ASW calculated using the peak data was significantly higher than that calculated using the gene activity data, with *P*-value of paired *t*-test being 1.12e-4 (Fig. 4b and Supplementary Fig. 12 in File “Supp Figures.doc”).

#### The introduction of semi-supervised training

For cross-omics label transfer, the RNA cell type labels are assumed to be part of the known information. scGCN and scJoint are the two methods among all the five methods that are designed for label transfer that are able to utilize this information through a semi-supervised training strategy. There have also been studies arguing that using available cell labels could achieve better cell embeddings (Dong and Alterovitz 2021; Xu et al. 2021; Chari et al. 2023). Therefore, we investigated whether introducing semi-supervised strategy to some of the joint embedding methods could increase the prediction accuracy.

From Fig. 4c and Supplementary Fig. 13 in File “Supp Figures.doc,” we observed that for metrics assessed on common cells, scDML was the only method that benefitted largely and significantly from semi-supervised training, while for methods including GLUE (multi), StabMap (multi), and GLUE, their prediction accuracy decreased significantly after using semi-supervised training. The weighted accuracy on ATAC-specific cells did not change much on any of the methods. For F1 of entropy and enrichment calculated using ATAC-specific cells, seven methods (MultiVI, scJoint, GLUE, SCALEX, scDART, scDML, and cross-modal AE) had decreased values and StabMap was the only method that had increased values. Methods that were insensitive to the introduction of semi-supervised training were Cobolt, scVI, uniPort, and StabMap.

### Time and memory usage comparison

We used mMOp to assess the scalability of all the 27 methods, because this was the only task that originally contained more than 50k cells for both the RNA and the ATAC data and had corresponding paired data. Specifically, we ran each method on a series of data sizes, including 1k, 3k, 5k, 10k, 15k, 20k, and 50k cells, and recorded the running time and peak CPU memory usage.

**Fig. 5.**
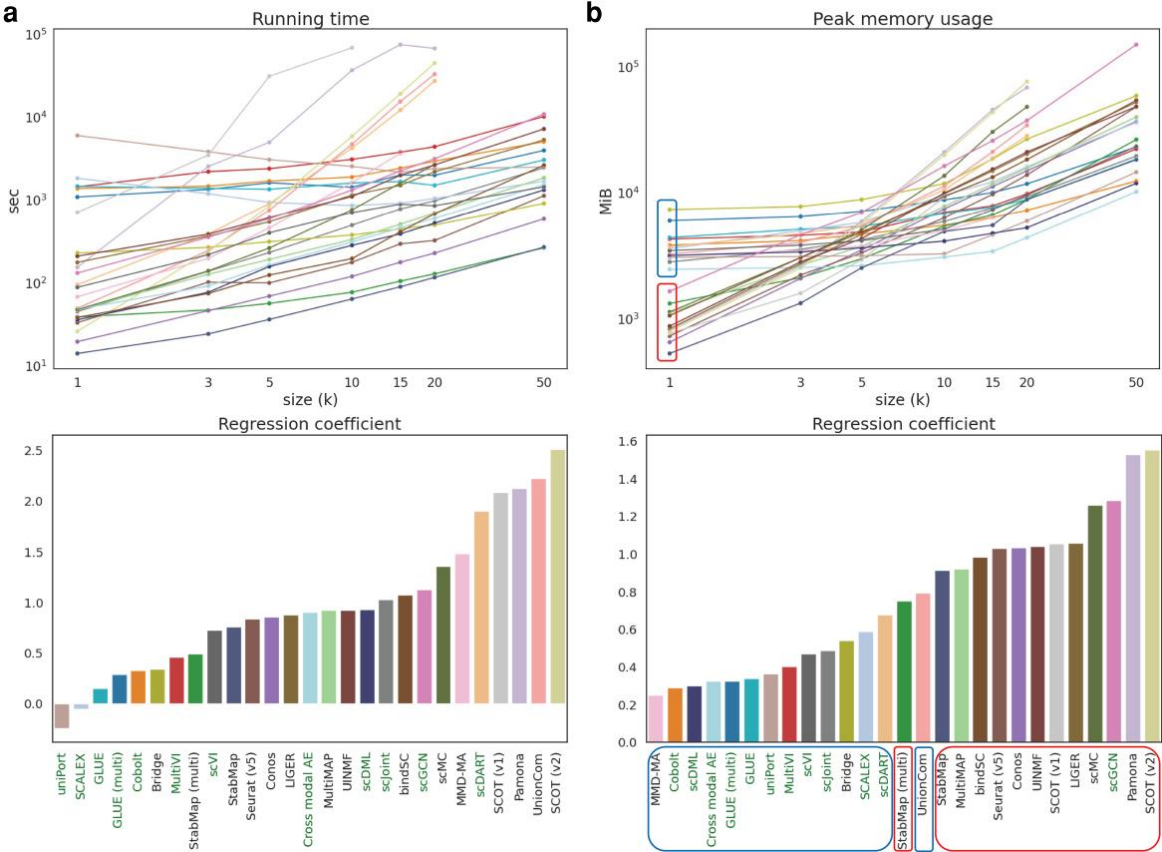
Computational efficiency comparison among 27 methods across seven data sizes. (a) Running time comparison. (b) Peak memory usage comparison. The lineplot at the top of each subfigure shows the trends of the corresponding computational efficiency metric of all methods across increasing data scale. Both the *y*-axis and the *x*-axis are in log scale. The barplot at the bottom presents the coefficient of each method's log-scaled metric fitted using linear regression on log-scaled data size. The color code for each method is consistently used in all plots. Methods whose names are in green are deep-learning-based methods. For (b), methods whose lineplots are marked by a red square at size = 1k are also highlighted by red squares along the *x*-axis of the barplot. So as for the methods whose lineplots are marked by a blue square.

As shown in Fig. 5a, the top five methods that increased fastest in running time as the data size increased were SCOT (v2), UnionCom, Pamona, SCOT (v1), and scDART. The first four methods were designed based on optimal transport or metric space matching that involved time-consuming matrix optimization especially when data scale went up. scDART is a deep-learning-based method, but it requires the calculation of pairwise distances between cells in the latent space. The top five methods that scaled slowest as the data size increased were uniPort, SCALEX, GLUE, GLUE (multi), and Cobolt. All of them are deep-learning-based methods, but unlike scDART, none of them involved the computation of loss terms that scaled non-linearly with the data size. The first four methods even employed early stopping and iteration-based training instead of epoch-based training to control the running time (see comparisons among deep learning-based methods in Table 2).

**Table 2.** A summary of common hyperparameters of deep-learning-based methods.

	Default/used epochs	Default/used batch size	Early stopping?	Optimizer	Learning rate
scGCN	200	N^a^	√	Adam	0.01
scJoint	20^b^	256^b^	X	SGD	0.01^b^
scDML	50	64	X^c^	Adam	0.01
Cross-modal AE^d^	100	128	X	SGD	0.005
GLUE	max(48/0.002/(N/batch size), 48)^e^	128	√	RMSprop	0.002
scDART	500^f^	128	X	Adam	0.0003
SCALEX	30,000/(N/batch size)^e^	64	√	Adam	0.0002
uniPort	30,000/(N/batch size)^e^	256	√	Adam	0.0002
scVI/MultiVI	min(20,000/N*400, 400)	128	X^c^	Adam	0.001
Cobolt	100	128	X	Adam	0.001^f^

^a^For the entire table, we use *N* to indicate the total number of cells.

^b^For scJoint, there were no default numbers of epochs, batch size or learning rate provided. The values shown in the table were what we used as default in this study, which were chosen by referring to the values used in its real data applications. Note that for the learning rate, we used 0.001 for mMOp (both stage 1 and 3), HSPC [paired] and mEmbryo [paired] (only stage 3) since 0.01 would cause the training of scJoint to diverge on these tissues.

^c^By default early stopping was not used for scDML, scVI and MultiVI, but users can switch that on if desired.

^d^For cross-model AE, there were no code provided for this algorithm from the original authors. To implement it, we downloaded the version provided by a comment paper (Xu and McCord 2022).

^e^For GLUE, SCALEX and uniPort, instead of fixing the number of epochs, they set the maximum number of iterations, which is defined as training one batch size samples.

^f^Although the default number of epochs is 700 as written in its training function, we used 500 because this was the number used in the original publication and its online tutorial.

^g^For Cobolt, we adjusted the learning rate down to 0.0002 for mEmbryo in all experiments and for mBrain (snap) and mKidney in the data binarization experiment because we found otherwise the training would diverge.

From Fig. 5b, we can observe that the top five methods that had the fastest increase in peak CPU memory usage were SCOT (v2), Pamona, scMC, scGCN, and LIGER. Among them, scGCN is a deep learning-based method, but it involved a data processing step where both intra-data and inter-data graphs were constructed that consumed a lot of memory. The top five methods that scaled slowest as the data size increased were MMD-MA, Cobolt, scDML, cross-modal AE, and GLUE (multi), among which the last four are all deep learning-based methods and all of them used GPU for training. We found that if we divided all methods into two groups based on their peak memory usage at data size 1k (Fig. 5b, lineplot), all methods from the blue group except UnionCom were the top methods that scaled slowest with the data size (Fig. 5b, barplot). This suggests that the memory usage at a small data size was related to the increasing rate of a method's memory usage when the data scaled up. Methods that consumed more memory at a relatively small sample size (1k) usually scaled better with larger data. Moreover, we found all methods except Bridge utilized GPU and Bridge relied on a technique called dictionary learning that is inherently scalable to large datasets (Hao et al. 2024).

Heatmap view of running time and memory usage comparison across all methods and data sizes can be found in Supplementary Fig. 14 in File “Supp Figures.doc.”

## Discussion

We performed a comprehensive benchmarking study on 27 computational tools that can be used to perform scATAC-seq label annotations, where five tools can directly perform label transfer from scRNA-seq to scATAC-seq data and 22 tools were originally designed for joint embedding (label transfer via training an additional kNN classifier), across 27 tasks composed of single-cell RNA and ATAC data collected from both human and mouse tissues. Moreover, we studied the impact of the data properties (data imbalance and cross-omics cell type dissimilarity) and method properties (utilization of paired data, peak information, and semi-supervised training strategy) on the performance of different methods. Prediction accuracy was evaluated on common cells and ATAC-specific cells separately. In addition, the running time and the peak CPU memory usage of each method were compared across a range of data sizes.

For the 18 tasks that did not involve additional paired data, the best performers in terms of prediction accuracy on common cells that were among the top three performers in more than half of the tasks were GLUE (16/18) and bindSC (13/18), followed by Seurat (v5) (6/18), MultiMAP (4/18), UINMF (4/18), scJoint (4/18), and uniPort (3/18). For the nine tasks that had available paired data from the same tissue, the methods that were among the top three performers in at least three tasks were Bridge, GLUE (multi), bindSC, and GLUE. For ATAC-specific cells, scGCN was the one among all 27 methods that had the most similar performance to a random classifier, which was hypothetically the best classifier for ATAC-specific cells. Moreover, we found that scJoint had high enrichment scores and weighted accuracy, indicating its tendency to misclassify unobserved cell types with high confidence. The methods that consistently performed poorly were MMD-MA, Pamona, UnionCom, SCOT (both v1 and v2), and StabMap. Except for StabMap, all these methods relied on cell–cell distance information and tried to align such information from RNA and ATAC without incorporating any prior information about the relationships between features from the two modalities, causing potential misalignment of cell types of similar abundance instead of similar biology. For StabMap, although it related each ATAC peak with its nearby gene, it only kept one ATAC peak if multiple peaks were related with a gene and treated the ATAC peak and the gene as the same feature. This aggressive strategy might cause the loss of useful biological information.

Based on the observations from the 27 tasks, we concluded that when paired data from the same tissue were available to be jointly integrated with unpaired data, the best methods were Bridge and GLUE (multi) that could use such paired data. For mKidney, mBrain (atlas), mBrain (snap), and mBrain (10x), neither Bridge nor GLUE (multi) ranked among the top two performers. The sequencing depths of their multimodal ATAC data were all relatively low with the median number of ATAC features per cell ranging from 570 to 1,086 (Supplementary Table 2 in File “Supp Tables.xlsx”). While for other tasks, the numbers were usually in thousands. Such high sparsity might be the reason why Bridge and GLUE (multi) performed relatively poorly on these tasks. When additional paired data were not available, the best performers were bindSC and GLUE and a common property of these two methods was that they both used the ATAC peak data instead of purely relying on gene activities. To align the feature sets between RNA gene-level data and ATAC peak-level data, GLUE utilized a prior knowledge graph that recorded the regulatory interactions among genes and ATAC peaks, and bindSC used a technique call bi-order canonical correlation analysis to align both the cells and features between RNA and ATAC data with gene activity matrix only used for algorithm initialization.

Even though GLUE (multi) and Bridge could utilize paired data and achieved the best performance on the third group of tasks, we found the utilization of paired data did not always improve the prediction accuracy, such as MultiVI vs scVI and StabMap (multi) vs StabMap. This might be due to some inherent limitations in the design of these algorithms. For example, MultiVI calculated the joint embedding from two modalities by just averaging the modality-specific embeddings and StabMap only used one dataset as reference and performed linear transformation to map other datasets to the reference.

Moreover, we found the introduction of semi-supervised strategy had a negative impact or no effect on most methods. The method that was most negatively impacted by this was GLUE. Semi-supervised training did increase the prediction accuracy of scJoint on average, but the change was not significant. The only significant and positive change was observed on scDML, which is a method based on deep metric learning by first finding similar and dissimilar clusters between RNA and ATAC data. Note that the semi-supervised training was realized through concatenating an additional layer of neural net as a classifier to the embedding layer of a tested method. Therefore, the observation that semi-supervision only worked on scDML might be because both are clustering-based techniques. This suggests that semi-supervised training is not always compatible with a method.

Apart from the choice of the computational tool, we found that the prediction accuracy can be affected by the properties of a dataset. Basically, datasets with large proportions of modality-specific cell types, high discrepancy in the common cell type compositions between modalities, and low cross-omics similarity in common cell types had poor accuracy scores across all the methods.

Almost all the methods were negatively impacted by the data imbalance even though some methods tried to deal with discrepancy in cell type distributions between two modalities through their unique designs, such as using mutual nearest neighbors (Barkas et al. 2019; Stuart et al. 2019; Song et al. 2021; Lin et al. 2022; Hao et al. 2024), reweighting cells (Cao and Gao 2022), unbalanced optimal transport (Cao et al. 2021; Demetci et al. 2022b; Cao et al. 2022), or integrative non-negative matrix factorizations (Welch et al. 2019; Kriebel and Welch 2022). Since we removed imbalance at two levels by only removing modality-specific cell types and by making cell types perfectly balanced between RNA and ATAC, we were able to perform pairwise comparisons among these two experiments and the original results. We found some methods (e.g. scJoint, bindSC, UINMF, LIGER, and Conos) could only benefit from removing modality-specific cell types, which suggests their algorithms can deal with the imbalance in cell type compositions as long as cell types from two modalities are the same. Some methods (e.g. Bridge, GLUE, MultiVI, Seurat (v5), MultiMAP, scGCN, and SCALEX) could benefit further from balancing the cell type compositions between modalities. This implies that these algorithms cannot properly deal with data imbalance.

Although data binarization was adopted by scJoint as a potential way to reduce the difference in data distribution between two modalities, we found the prediction accuracy was negatively affected and the F1 of entropy and enrichment was positively affected by data binarization for most methods. We speculate that this was because binarization removed the differences among cell types within each modality and thus made it harder for algorithms to label cell types. Another potential reason was that some methods assumed that single-cell data followed some over-dispersed distributions like negative binomial distribution, so binarization could have caused the violation of this model assumption.

In terms of method scalability, we found the most time and memory-consuming methods are those that involved matrix optimization (e.g. SCOT, Pamona, and UnionCom) or pairwise distance calculation among all cells (e.g. scMC, scGCN, and scDART). In contrary, the most memory-efficient methods were usually the deep-learning-based ones (e.g. uniPort, SCALEX, GLUE, Cobolt, and MultiVI) that could use GPU and minibatch training strategy. The methods that consumed less time were also deep learning-based algorithms, especially those that adopted early stopping and fixed number of training iterations. Iteration-based training can largely save the running time compared to epoch-based training given a fixed minibatch size. This is because for an epoch all cells in the training data are used, while for an iteration only a minibatch of cells are used. Apart from deep learning-based method, we found Bridge was also both time and memory efficient because it utilized dictionary learning and only performed heavy computation on a subset of data.

Our study has some limitations. First, although we tried to collect as many data as possible for this benchmarking study, the types of tissues and sequencing technologies we covered were still limited, especially for tasks that had both unpaired data and corresponding paired data. Second, we used the cell type labels provided by their original publications as the ground truth. However, the task of cell type annotation is usually a combination of results from computational tools and manual adjustment by experts, so the derived labels could be biased and subjective. For some finer levels of cell type annotation, even researchers from the same field can have different opinions.

To summarize, we have the following suggestions for users to choose among currently available tools. If high quality paired data can be found to transfer labels from unpaired scRNA-seq to scATAC-seq data, Bridge and GLUE (multi) are likely the best method; otherwise, bindSC and GLUE are the recommended methods. Both Bridge and bindSC are methods written in R and compatible with the most popular single-cell R package Seurat, so R users should feel more comfortable to use these two methods. For Python users, GLUE can be a better choice not only because it is written in Python and compatible with Python's single-cell module Scanpy (Wolf et al. 2018), but also because this tool is able to run both with and without pairing information between two modalities. Note that none of these methods had a good performance on ATAC-specific cells. Therefore, if one cares about ATAC-specific cell types, a better strategy might be using scGCN first to identify potential cells unique to ATAC, and manual annotations can be performed on cells that have high entropy and low enrichment.

For future directions, we think an ideal method for label transfer from scRNA-seq to scATAC-seq should take the following aspects into consideration. First, such a method should thoroughly deal with the data imbalance between scRNA-seq and scATAC-seq data. Second, such a method should take care of the peak-level information instead of purely relying on gene activity matrix to represent the ATAC data. Third, it should be able to properly incorporate paired data if such data with high quality are available. Fourth, it should not over-normalize the original data, like through data binarization. Some studies have argued that the binarization of scATAC-seq data would suffer from loss of information (Martens et al. 2024; Miao and Kim 2024). Fifth, semi-supervised training strategy should be used with caution as this would not always benefit a method and even could have a negative effect. Last, future methods can borrow the idea from GLUE that includes prior knowledge to inform the relationships among features from different modalities.

## Supplementary Material

jkag026_Supplementary_Data

## Data Availability

All the single-cell data used in this study are publicly available. Detailed information of each data and their downloadable links can be found in Supplementary Table 1 in File “Supp Tables.xlsx.” The related scripts for reproducing results in this study are available at our GitHub repository: https://github.com/AprilYuge/ATAC-annotation-benchmark/tree/main/G3. Supplemental material available at G3 online.
