# Supplementary material for: A comprehensive benchmarking study on computational tools for cross-omics label transfer from single-cell RNA to ATAC data
Source: G3 (Bethesda). 2026 Feb 8;16(4):jkag026. doi: 10.1093/g3journal/jkag026 (PMC13042286; doi:10.1093/g3journal/jkag026)
Supplement: jkag026_Supplementary_Data [file jkag026_supplementary_data.zip › Supp_Text_G3-2025-406310.docx]

**Text S1.** **Data Processing Description by Dataset**

The following text provides supplementary information for the “Single-cell data preprocessing” section in Materials and Methods.

Human T-cell-depleted bone marrow (TDBM) (paired). There was no discrepancy between the analysis pipeline we performed for this tissue and the one described previously.

Human kidney (unpaired). There was no discrepancy between the analysis pipeline we performed for this tissue and the one described previously.

Human fetal atlas (unpaired). There was no discrepancy between the analysis pipeline we performed for this tissue and the one described previously. For this tissue, the raw gene activity matrix was available online.

Human basal cell carcinoma (BCC) (unpaired). There was no discrepancy between the analysis pipeline we performed for this tissue and the one described previously.

Human heart (unpaired). There was no discrepancy between the analysis pipeline we performed for this tissue and the one described previously.

Human PBMC COVID (unpaired). For the scATAC-seq data, although the original publication claimed that the reference genome they used was hg19, it was actually hg38 because only hg38 could yield correct TSS enrichment score. Therefore, we used hg38 to calculate gene activities for ATAC.

Human PBMC. The reference genomes used for the unimodal ATAC (hg19) and the multimodal ATAC (hg38) data were different and only the latter had public raw sequence data in fastq formats. We remapped the multimodal data using cellranger-arc to hg19 to get the peak count matrix and fragment files to align peak sets between the unimodal and the multimodal ATAC data.

Human BMMC. This is so far the largest single-cell multimodal RNA and ATAC dataset with well-annotated labels and hierarchical batch structures. To mimic the case where scRNA-seq, scATAC-seq and multimodal data were measured separately, we manually separated all batches to three groups without any overlaps. Specifically, batches s1d2, s1d3, s3d3, s4d9, and s3d10 were used as scRNA-seq (26,450 cells), s2d4, s2d5, s3d6, s3d7, and s4d8 were used as scATAC-seq (24,332 cells), and s1d1, s2d1, s4d1 were used as multimodal data (18,467 cells).

Human HSPC. For the unimodal ATAC data, we didn't use Signac to calculate the gene activity matrix because we couldn't find any accompanying fragment files which were required by Signac to do the calculation. Therefore, we wrote a function called 'CreateGeneActivityMatrix' (can be found in our online GitHub [repository](https://github.com/AprilYuge/ATAC-annotation-benchmark/tree/main/G3)) that can calculate gene activities purely rely on the peak count matrix by summing up peak counts within the 2000 base pairs around the transcription starting site of a gene. For the multimodal ATAC data, the gene activity calculation was still performed by default using Signac because fragment files were available. Moreover, since the original reference genomes used for the unimodal ATAC (hg19) and the multimodal ATAC (hg38) data were different and no raw fastq files were found, we lifted peaks of the unimodal ATAC data over from hg19 to hg38 using UCSC liftover utility for peak set alignment.

Mouse skin (paired). There was no discrepancy between the analysis pipeline we performed for this tissue and the one described previously.

Mouse retina (paired). For the raw gene activity matrix, we used the one provided by the original publication, which was calculated by Cicero.

Mouse spleen (unpaired). For the raw gene activity matrix, we used the one provided by the original publication, which was calculated by snapATAC.

Mouse endothelial (unpaired). There was no discrepancy between the analysis pipeline we performed for this tissue and the one described previously.

Mouse atlas (unpaired). There were two types of RNA data: one was sequenced by scRNA-seq (droplet) and the other was sequenced by SMART-seq (FACS). We processed and benchmarked these two types of RNA data separately with the same ATAC data. For the ATAC data, we used the gene activity matrix provided by the original publication.

Mouse brain. We had three pairs of unimodal RNA and ATAC data for mouse brain. The first pair was composed of the brain cells from the previous mouse atlas data. For the RNA data, only the FACS data were used because the droplet data didn't have any brain cells. For the rest two pairs, they shared the same RNA data sequenced by Drop-seq. The two unimodal ATAC data were from separate studies, one sequenced by scATAC-seq and the other sequenced by snATAC-seq. For the former one, we used the default method (Signac) to calculate the gene activity matrix; for the latter one, we used the provided raw gene activity matrix calculated by snapATAC. All three pairs of unimodal data shared the same multimodal data from SNARE-seq. Since the reference genome used for the mouse atlas ATAC data (mm9) was different from the one used for the multimodal ATAC data (mm10), we lifted peaks of the mouse atlas brain ATAC data over from mm9 to mm10 using UCSC liftover utility for peak set alignment.

Mouse primary motor cortex (MOp). The raw gene activity matrix for the unimodal ATAC data was extracted from the snap objects provided by the original publication, which was computed using snapATAC.

Mouse embryo. For the unimodal ATAC data, although the fragment files were not available, we downloaded the fastq files and used the pipelines provided by the authors on GitHub (https://github.com/BPijuanSala/MouseOrganogenesis_snATACseq_2020) to generate a BAM file. After indexing the BAM file using SAMtools, we used sinto to generate a fragment file and then used tabix to sort, block-gzip compress ([bgzip](https://www.htslib.org/doc/bgzip.html)) and index the file.

Mouse kidney. For this tissue, we requantified the unimodal ATAC peaks on the multimodal peak set, because only the fragment files of the unimodal ATAC data were available. Moreover, like how we calculated the gene activities for human HSPC's unimodal ATAC data, we calculated gene activities for the multimodal ATAC data using the raw peak count data via 'CreateGeneActivityMatrix' because no fragment files were available.

**Text S2.** **Method Description.**

The following text provides supplementary information for the “Description and implementation of methods” section in Materials and Methods.

Conos. Conos is designed as a graph-based batch effect removal method. The joint graph embedding using nearest neighbors and Pearson correlation is constructed as the first step to connect all cells. Then, the label transfer from reference data to query data can be implemented by information propagation between graph vertices through an iterative diffusion process.

Seurat (v5). Seurat first identifies a set of anchors between the reference and the query data through canonical correlation analysis (CCA) and mutual nearest neighbors (MNNs). Then, a weight matrix is constructed to quantify the distance between each query cell and anchor cell in the query data by a Gaussian kernel. Last, the prediction score of any cell in the query data is calculated as a weighted average of labels of anchor cells in the reference data. Both the gene activity and raw peak count matrices are needed, with the former matrix required to obtain cross-omics anchors through CCA and the latter one recommended for nearest neighbor construction among the ATAC cells.

scGCN. The first step of scGCN is to build a hybrid graph of all cells using MNNs approach and CCA. Based on the constructed graph, a semi-supervised graph convolutional neural network is trained to embed cells from both reference and query data on the same latent space and predict cell type labels for cells in the query data.

scJoint. Like scGCN, is a semi-supervised neural network trained to jointly embed cells from both scRNA-seq and scATAC-seq. Different from scGCN that directly utilizes the trained network to predict probability vectors through Softmax layers, scJoint performs label transfer by training an additional kNN classifier in the embedding space. The loss function of scJoint is composed of a dimensionality reduction loss, a cosine similarity loss and a cross-entropy loss (calculated only using scRNA-seq data and the known cell labels). The role of the cosine similarity loss is to maximize the similarity between best aligned RNA and ATAC cells in the latent space. To account for the case where RNA and ATAC do not share the same cell types, only the top 80% of cells with the highest cosine scores are used to calculate this loss term.

Bridge. This method utilizes multimodal data as a bridge to transfer labels from scRNA-seq to scATAC-seq. The multimodal dataset is treated as a dictionary and each cell is an atom, on which dictionary representations of both unimodal scRNA-seq and scATAC-seq are constructed. After dimensionality reduction of multimodal cells via Laplacian Eigendecompostions, unimodal cells can be embedded on the same space by the dictionary representations. Then, the final label transfer can be achieved by any single-cell integration techniques and Bridge chooses mnnCorrect.

*All methods below are joint embedding methods for single-cell multi-omics data that do not directly perform label transfer from RNA to ATAC.*

MMD-MA. This is a manifold alignment method for unpaired single-cell multi-omics datasets based on maximum mean discrepancy (MMD). It aligns and embeds two datasets in Reproducing Kernel Hilbert spaces by minimizing the MMD across datasets. It does not require any overlap between the feature space of two datasets.

UnionCom. This method is based on metric space matching to jointly embed unpaired single-cell multi-omics datasets to a latent space. Specifically, it aligns cells across omics by matching the distance matrices by matrix optimization. Like MMD-MA, it does not require any correspondence information among features of the two datasets, so gene activity calculation is not needed.

SCOT. SCOT is an unsupervised alignment tool based on optimal transport (OT) for unpaired single-cell multi-omics data integration and it does not require any correspondence information among either cells or features. As the first step, an intra-domain distance matrix is calculated for each data by constructing a kNN graph. Then, cell-to-cell correspondence probabilities are derived based on Gromov-Wasserstein OT to align datasets cross omics. There are two versions of SCOT, for version 1, it cannot handle the situation where two omics have disproportionate cell type representations as it is based on global OT; For version 2, SCOT is extended to handle such case using unbalanced OT. Another difference is that version 1 integrates data without changing the number of input dimensions, but version 2 embeds data by projecting them to a joint latent space.

Pamona. Like SCOT, this method also relies on OT and is a partial Gromov-Wasserstein-based manifold alignment algorithm that can handles differences in cell type compositions between two modalities by allowing only a fraction of the total mass to be transported.

MultiMAP. This algorithm is designed based on the idea of UMAP to integrate and embed single-cell multi-omics datasets to a two-dimensional space. It first calculates both the intra and inter omics geodesic distances among cells and then constructs a multi-omics neighborhood graph based on these distances. Finally, it projects the data into a low-dimensional space by minimizing the cross-entropy of the graphs in the latent space and the manifold space. Both original peak data and gene activity data from ATAC are used to calculate intra and inter omics cell distances respectively.

scVI. This is a deep learning method based on a variational autoencoder (VAE) to integrate scRNA-seq data from different batches. Batch information is provided as a categorical variable to both the encoder and decoder to help derive a batch-free latent embeddings of cells from all datasets. Here, we applied scVI to jointly embed unpaired scRNA-seq data and scATAC-seq data's calculated gene activity matrix.

Cross-modal AE. This is an autoencoder-based method for unpaired multimodal data integration and embedding. After projecting data into a latent space using modality-specific encoders, data are aligned through adversarial training by connecting a discriminator to the latent space.

SCALEX. Like cross-modal AE, SCALEX is a deep learning method based on a VAE for single-cell data integration and can be applied to jointly embed single-cell RNA and ATAC data if gene activity matrix is calculated from the scATAC-seq data. It projects data into a batch-invariant latent space by using a batch-free encoder and a batch-specific decoder.

scDML. This method is also a deep learning method but utilizes deep metric learning to remove batch effects among datasets in a latent space projected through an encoder. Initial clusters are inferred by algorithms like Louvain before training a neural network and both intra and inter data cluster similarities are assessed by computing the number of kNN and MNN pairs, respectively. Deep metric learning is used to ensure that similar clusters among datasets are merged and unsimilar clusters are kept away from each other in the latent space.

uniPort. This method can perform joint embedding of unpaired single-cell multi-omics datasets by combining a coupled VAE and minibatch unbalanced OT. It utilizes the information from both common highly variable genes and data-specific genes by incorporating multiple decoders.

scDART. This is a scalable deep learning method that is designed specifically for integrating unpaired scRNA-seq and scATAC-seq data in a low-dimensional space. Unlike most methods, scDART doesn't require a pre-calculated gene activity matrix for the ATAC data. Instead, it introduces a non-linear gene activity function module that connects ATAC peaks (first layer) with RNA genes (second layer) and the weight matrix of this module is learned by putting a binary mask on it. The mask matrix is derived based on the genomic locations of peaks and genes. Additionally, scDART utilizes MMD as a kernel-based discrepancy to align similar cells across modalities in the latent space.

GLUE. Like some previous deep learning-based methods, GLUE is based on VAE, but the difference is that in addition to using omics-specific VAEs to learn cell embeddings, GLUE incorporates a knowledge-based graph VAE that utilizes prior information about the regulatory interactions among genes and ATAC peaks. Therefore, GLUE is immune to the information loss by avoiding the calculation of gene activities and the cross-omics joint embeddings can be guided by the prior knowledge graph. Moreover, a discriminator is used to align cell embeddings from different omics through adversarial training. To deal with the case where cell type compositions differ among omics, GLUE introduces weighted adversarial alignment that assigns weights to cells to balance cell distributions across omics. GLUE also has a extend version that can be used when both paired and unpaired data are available by penalizing distances between paired cells in the latent space.

scMC. This method relies on variance analysis to deconvolutes technical and biological variations among different single-cell datasets at the cell cluster levels. It starts with the identification of putative cell clusters for each data and then the inference of shared clusters between two datasets. Variance analysis is used to derive the correction vectors for batch effect removal.

bindSC. This method is based on bi-order canonical correlation analysis (CCA) for unpaired single-cell multi-omics data integration. CCA is performed iteratively to align both cells and features from two modalities. bindSC requires both the original peak matrix and calculated gene activity matrix from scATAC-seq data, but the gene activity matrix is only used to initialize the feature matching.

LIGER. This method relies on integrative non-negative matrix factorization (iNMF) to integrate and embed unpaired single-cell multi-omics datasets by delineating shared and data-specific features (metagenes). This method requires sharing features across datasets, so gene activity calculations are needed when integrating scATAC-seq and scRNA-seq data.

UINMF. UINMF is an extension of LIGER for the case where features are partially shared across datasets (but there must exist a set of features shared across all datasets). The key idea is to let data-specific features to inform the factorization by introducing an additional metagene matrix. To apply UINMF, both the original peak data and gene activity data from ATAC should be provided along with the RNA gene data so that both unshared and shared features from ATAC can be used to inform the joint embedding.

MultiVI. MultiVI is built based on scVI that can integrates both paired and unpaired scRNA-seq and scATAC-seq data so that gene activity calculations are not needed. The distance between two modalities is penalized through minimizing the KL divergence between their distributions in the latent space and the joint embeddings are calculated as the average of the embeddings from two modalities.

Cobolt. Like MultiVI, Cobolt is a multimodal VAE (MVAE) framework that can be used to jointly embed unpaired single-cell RNA and ATAC data by using additional paired multi-omics data. To better align data from different modalities in the latent space, Cobolt first fits the MVAE model and then uses the paired data to train cross-omics predictors that can correct for missing modalities of the unpaired data.

StabMap. StabMap is designed for mosaic data integration like UINMF where features are partially overlapped. However, it is different from UINMF and is more generalized in the sense that no set of features shared across all datasets is required. The only requirement is that there is a way to draw a path from every data to every other data and two data are connected if there exist shared features. Therefore, StabMap can be used to integrate unpaired scATAC-seq and scRNA-seq data with paired data without calculating gene activities. According to the original paper, StabMap can also be applied to unpaired data only, by linking ATAC peaks with nearby genes and treat them as the same features.

**Text S3.** **Evaluation Metrics Description**

The following text provides supplementary information for the “Evaluation metrics” section in Materials and Methods.

Overall accuracy. After getting the predicted probability matrix across all cells in scATAC-seq, the cell type that had the highest predicted probability was assigned to each cell as the predicted label. Then the overall accuracy was calculated using the predicted labels and true labels for all common cells. We didn't calculate this metric for ATAC-specific cells because it would be exactly zero.

Balanced accuracy (macro recall). The overall accuracy can be easily affected by the composition of cell types in data. To remove that influence and treat all cell types equally, we calculated balanced accuracy for all common cells, which is the average of recalls for each cell type (macro recall),

$BACC=\sum_{c\in C} \frac{T_{c}}{N_{c}},$ (1)

where $C$ is the set of all common cell types, $N_{c}$ is the number of cells in cell type $c$, and $T_{c}$ is the number of cells correctly predicted as cell type $c$.

F1 score. Precision is defined as true positive (TP) over the summation of TP and false positive (FP) and recall is defined as TP over the summation of TP and false negative (FN). F1 score is the harmonic mean of precision and recall,

$F_{1}=2\frac{Precision\cdot Recall}{Precision + Recall}$. (2)

Since this is a multi-class classification problem, we need to specify whether we want macro or micro level metrics. It is easy to show that overall accuracy is equivalent to micro precision, recall and F1 score under the multi-class scenario. Therefore, we calculated macro level precision and recall in this study, which is the average of precisions and recalls obtained for each class. Then, macro F1 score is calculated based on macro precision and recall. Like overall accuracy and balanced accuracy, F1 score was calculated only for common cells.

Weighted accuracy. To account for the prediction uncertainty and similarity across cell types. We proposed a weighted accuracy (WACC) by taking the average of the predicted probability vector weighted by cell type similarities:

$WACC=1/N\sum_{i} \sum_{j\in C_{R}} S_{c(i),j}P_{i,j}$. (3)

In the equation above, $P$ is the predicted probability matrix with each row as a cell in scATAC-seq and each column as a cell type observed in scRNA-seq reference data. $C_{R}$ is the set of all cell types in scRNA-seq and $N$ is the total number of scATAC-seq cells. $S$ is a cross-modality cell type similarity matrix with each row as a cell type in scATAC-seq and each column as a cell type in scRNA-seq and $c(i)$ is a function mapping cell $i$ to its true cell type label. Since for ATAC-specific cell types, their similarities to cell types in RNA data could be assessed through common cell types and recorded in the cross-modality similarity matrix $S$, weighted accuracy was calculated on both common cells and ATAC-specific cells.

The similarity matrix was calculated in three steps. First, partition-based graph abstraction (PAGA) [1] was performed on the normalized count matrix of scRNA-seq and gene activity matrix of scATAC-seq separately. Then, the within-modality similarity matrix was calculated based on the Euclidean distance of each pair of cell types using the PAGA positions. For cell types $i$ and $j$, their within-modality similarity was calculated as:

$S_{i,j}^{mod}=exp(-||PAGA_{i}^{mod}-PAGA_{j}^{mod}||)$*,* $mod\in[ATAC,RNA]$*.* (4)

Last, we calculated the cross-modality similarity matrix using the two within-modality matrices by considering three scenarios. If two cell types existed in both modalities, their similarity was calculated as the average of two within-modality similarities:

$S_{i,j}=1/2(S_{i,j}^{ATAC}+S_{i,j}^{RNA})$*,* $i,j\in[common cell types]$*.* (5)

If one cell type is modality-specific, its similarity with any common cell type would be the similarity calculated using the modality that contained the two cell types:

$S_{l,c}=S_{l,c}^{ATAC}$*,* $l\in[ATAC-specific cell types],c\in[common cell types]$*,* (6)

$S_{c,k}=S_{c,k}^{RNA}$*,* $k\in[RNA-specific cell types],c\in[common cell types]$*.* (7)

If a cell type $l$ only existed in scATAC-seq and the other cell type $k$ was only observed in scRNA-seq, their similarity was calculated as

$S_{l,k}=[{S^{ATAC}}_{l, common}\circ1{{(S}^{ATAC}}_{l, common}\geq.5){]S^{RNA}}_{common, k}/\sum_{i\in common} 1{{(S}^{ATAC}}_{l, i}\geq.5)$, (8)

where $S^{ATAC}$ and $S^{RNA}$ are within-modality similarity matrix for ATAC and RNA, respectively, $1$ represents an indicator function and $common$ is the set of all common cell types. The first product is Hadamard product which is element wise and the second product is matrix multiplication. This metric was calculated for both common cells and ATAC-specific cells separately.

Entropy and enrichment. To further evaluate the performance of methods on ATAC-specific cell types, we borrowed the two metrics proposed in scGCN which are scaled entropy and enrichment [2]. Scaled entropy is defined as

$NE=-\frac{1}{Mlog_{2}|C_{R}|}\sum_{i} \sum_{j\in C_{R}} \frac{S_{i, j}}{\sum_{j\in C_{R}}S_{i, j}}log_{2}\frac{S_{i, j}}{\sum_{j\in C_{R}}S_{i, j}}$, where $S_{i, j}=\frac{P_{i, j}}{Q_{j}}$. (9)

$P_{i, j}$ is the predicted probability for cell $i$ with unique cell type label in scATAC-seq and cell type $j$, and $Q_{j}$ is the proportion of cell type $j$ in scRNA-seq as the background probability. $C_{R}$ is the set of all cell types in scRNA-seq and $M$ is the total number of scATAC-seq cells with unique cell labels. The final score is normalized by $log_{2}|C_{R}|$ to make it in the range of [0, 1]. Another metric is enrichment score,

$ES=\frac{1}{M}\sum_{i} max_{j\in C_{R}}\frac{S_{i, j}}{\sum_{j\in C_{R}}S_{i, j}}$. (10)

The enrichment score is also bounded within 0 and 1. For cell types only observed in scATAC-seq, an ideal method should deliver high normalized entropy and low enrichment score. Therefore, we also calculated an F1 score to combine these two

$F_{1}=2\frac{NE\cdot(1-ES)}{NE + (1-ES)}$. (11)

Running time and memory. For methods that require GPUs as indicated in Table 1, they were run using GPUs; and for the rest methods, they were run using CPUs. The CPU of our device is Intel ® Xeon ® Gold 6240, 2.6 GHz, and the GPU is NVIDIA A100 with 40 GB RAM. When evaluating running time, we did not count the time used for data preprocessing (e.g. remaping to alternative reference genome, requantifing scATAC-seq peaks, and calculating gene activity matrix) because the needed steps for different tissues were different. For memory assessment, we used the peak memory usage to compare across different methods. To obtain the peak memory usage, we used 'memory_profiler.memory_usage' for Python methods and peakRAM for R methods.

**References**

[1] F. A. Wolf *et al.*, "PAGA: graph abstraction reconciles clustering with trajectory inference through a topology preserving map of single cells," *Genome biology,* vol. 20, no. 1, pp. 1-9, 2019.

[2] Q. Song, J. Su, and W. Zhang, "scGCN is a graph convolutional networks algorithm for knowledge transfer in single cell omics," *Nature communications,* vol. 12, no. 1, p. 3826, 2021.
